# Exosomes and their distinct integrins transfer the characteristics of oxaliplatin- and 5-FU-resistant behaviors in colorectal cancer cells

**DOI:** 10.1186/s10020-025-01110-y

**Published:** 2025-02-06

**Authors:** Zeynab Vakili-Ghartavol, Hoda Deli, Amir Shadboorestan, Roxana Sahebnasagh, Elahe Motevaseli, Mohammad Hossein Ghahremani

**Affiliations:** 1 Department of Molecular Medicine, School of Advanced Technologies in Medicine, Tehran University of Medical Sciences, Tehran, Iran; 2 Department of Toxicology, Faculty of Medical Sciences, Tarbiat Modares University, Tehran, Iran; 3 Department of Toxicology and Pharmacology, Faculty of Pharmacy, Tehran University of Medical Sciences, Tehran, Iran

**Keywords:** ITG, Exosome, Drug resistance, 5-FU, Oxaliplatin, Colorectal cancer

## Abstract

**Background:**

Exosomes are communication carriers and suitable biomarker candidates due to their cargoes with specific dynamic profiles. Integrins, as valuable prognostic markers in cancer, have importance in exosome-cell interaction. However, the role of exosome integrins in chemoresistant colorectal cancer remained unclear.

**Methods:**

Oxaliplatin- and 5-FU-resistant cells (OXR and FUR) were established from human HCT-116 cells of colorectal cancer. Exosomes were collected from untreated and treated cells with oxaliplatin or 5-FU. Exosomes were isolated via ultracentrifugation and characterized using DLS and electron microscopy to evaluate size and morphology. Western blot analysis was employed to identify exosomal markers. The effects of exosomes on parental cells were examined using various methods, including MTT assay for proliferation, wound healing assay for migration, flow cytometry for cell cycle and apoptosis analysis, Matrigel-coated transwell inserts for invasion, and western blot for integrin expression evaluation.

**Results:**

Exosome integrins determine resistance behaviors in cells. We observed that exosomes from OXR cells or OXR cells treated with oxaliplatin increased ITGβ3 expression and decreased ITGβ4 expression in parental cells, resulting in distinct resistance behaviors. Exosomes from FUR cells or FUR cells treated with 5-FU reduced ITGβ4 levels and elevated ITGαv levels in parental cells, leading to varying degrees of invasive resistance behaviors. These findings suggest that exosome integrins may affect these behaviors. High ITGβ3 exosomes induced oxaliplatin resistance behaviors in parental cells. Lowering ITGβ3 levels in these exosomes inhibited the resistance behaviors observed in these cells. FUR exosomes that overexpressed ITGαv or ITGβ4 resulted in invasive 5-FU resistance behaviors in parental cells. A reduction in these exosome integrin levels led to moderate invasive behaviors. The decrease of ITGβ4 in FUR cell exosomes inhibited resistant migration and proliferation in parental cells. A twofold reduction of ITGαv in FUR cell exosomes resulted in a threefold decrease in invasion and inhibited migration in parental cells compared to those treated with high ITGαv exosomes.

**Conclusion:**

Our findings reveal that, despite discrepancies between cellular integrin patterns and cellular behaviors, the levels of exosomal ITGβ3, ITGαv, or ITGβ4 could serve as potential diagnostic and therapeutic markers for resistance to oxaliplatin and 5-FU in future cancer treatments.

**Supplementary Information:**

The online version contains supplementary material available at 10.1186/s10020-025-01110-y.

## Introduction

Drug resistance has often remained a principal obstacle to cancer therapy, particularly colorectal cancer (CRC), treatment failures continue to cause poor survival in CRC patients, which exhibited the second-highest fatality rate among all cancers (9.2%) (Van der Jeught et al. 2018; Vasan et al. 2019; Xiao et al. 2020). Oxaliplatin and 5-fluorouracil (5-FU) are used in the first line of treatment protocol in advanced CRC patients (Gu et al. 2019; Azar et al. 2021). However, resistance to both drugs due to multiple mechanisms frequently occurs (Li et al. 2022). Integrins as adhesion receptors are pivotal protein molecules in cell signaling pathways controlling cell fate such as survival, proliferation, migration, invasion, angiogenesis, metastasis, and drug resistance with often overexpressed patterns and distinct functions in various cancers. The prominent roles of integrins in drug resistance have led to cancer treatment failures, where nowadays several clinical trial studies have investigated integrins as therapeutic targets for inhibiting the signaling pathways aberrantly activated (Liu et al. 2023; Hamidi and Ivaska 2018; Seguin et al. 2015). However, thus far, most integrin-targeted drugs have failed in phase III clinical trials. Clinical trials with inhibitors of α5β1, αvβ3, and αvβ5 integrin have not shown satisfying effects in cancer patients, and drug resistance has been repeatedly reported (Liu et al. 2023). Notably, drug discovery programs were initiated with uncontrolled excitement before a proper understanding and investigation of integrins. Therefore, clinical trials were further complicated by integrin complex pathophysiology. (Cox 2021). However, integrins remain promising therapeutic targets due to their dual critical roles in regulating the intrinsic growth of tumor cells and modulating the immune responses in the tumor microenvironment (Liu et al. 2023).

Exosomes are essential mediators in cell-cell communication. These extracellular nano-vesicles (30–200 nm) (Xiao et al. 2020) are uptake by recipient cells, transferring their cargoes and affecting the phenotype of recipient cells (Wortzel et al. 2019; Jena and Mandal 2021). Tumor-derived exosomes can remodel the microenvironment of secondary distant sites to become suitable for metastasis of tumor cells (Casari et al. 2021). They also play critical roles in drug resistance (Corcoran et al. 2012; Yin et al. 2019). Furthermore, many integrin types are packaged in tumor exosomes. Exosome integrins contribute to cancer cell colonization and the development of pre-metastatic niches (Mashouri et al. 2019). Preferential packaging of particular integrins in exosomes determines organs that uptake tumor exosomes where tumors metastasize. The integrins of α6β4 and α6β1 were enriched in breast cancer exosomes that metastasize to the lung organ, while ITGαvβ5 was abundant in pancreatic cancer exosomes that metastasize to the liver (Hoshino et al. 2015). Interestingly, exosomal proteins exhibit a multifaceted and dynamic profile and can be potential candidates for biomarkers. Numerous studies reported protein exosomes as potential biomarkers, such as high levels of exosomal S100A4 in metastatic hepatocellular carcinoma and pancreatic cancer, overexpression of exosomal FMNL2 in CRC that induces CRC angiogenesis and metastasis (Zhong et al. 2024), overexpressed exosomal heat shock protein DNAJB8 in oxaliplatin-resistant SW-480 and SW-620 colon cancer cell lines (Wang et al. 2022), and exosomal p-Stat3 in RKO cells of colon cancer resistant to 5-FU (Kotelevets and Chastre 2023). However, the significance of exosome integrins in the chemoresistance of colorectal cancer remained unclear.

In this study, we investigated ITGαv, ITGβ1, ITGβ3, and ITGβ4 expression patterns in exosomes from oxaliplatin- and 5-FU-resistant and parental cells as well as the treatment effects of 5-FU and oxaliplatin on the integrin expression patterns of these exosomes. We also examined the impact of these exosomes on secondary cell behaviors.

## Materials and methods

### Cell line and culture

The human colorectal carcinoma HCT-116 cell line was purchased from the Pasteur Institute (Tehran, Iran). The cells were cultured in 70% RPMI1640 plus 30% DMEMF12 (Biowest, UK) with 10% Fetal Bovine Serum (FBS) (Biowest) and 1% Penicillin-Streptomycin solution (Biowest) and maintained under 37^0^C and 5% CO2 incubation.

### Establishment of oxaliplatin- and 5-FU-resistant cells

Oxaliplatin- and 5-FU-resistant HCT-116 cell lines were established with stepwise increased concentrations of drugs. HCT-116 cells were first exposed to an initial 0.5 µM oxaliplatin and 5 µM 5-FU (lower than the 72-h IC_50_) separately in complete media. Because of stability, 5-FU was added daily to the culture medium, and cells remained exposed for 3–4 days. Then, cells were maintained in a drug-free medium to grow until 70–80% confluence. The cells were passaged and exposed to the same drug concentration to generate stable cells, repeating processes more than twice. Then, the drug concentration was increased, and methods were performed for oxaliplatin with 1, 5, 7, 10, 15, and 30 µM, and 5-FU with 1, 10, 15, and 40 µM. Finally, the 5-FU-resistant cells (FUR) surviving in a 40 µM dose of 5-FU and the oxaliplatin-resistant cells (OXR) in a 30 µM dose of oxaliplatin were established. The established OXR and FUR cells were maintained in a drug-free medium. The 72-h IC_50_ of 5-FU and oxaliplatin was evaluated in these cells.

### Collection of cell culture-conditioned media (CM)

First, parental (P) and oxaliplatin- and 5-FU-resistant cells were separately seeded into 175-cm^2^ cell culture flasks with a complete medium and incubated at 37^0^C under 5% CO2. Attached cells with 70% confluency were washed twice with PBS and placed in the free-FBS-conditioned medium for 48–72 h. Then, the cell culture supernatants were harvested to obtain exosomes of parental (EXO-P), oxaliplatin-resistant (EXO-OXR), and 5-FU-resistant (EXO-FUR) cells. To generate EXO-P5, EXO-P30, EXO-OXR5, and EXO-OXR30, cell supernatants collected from P and OXR cells cultured in 1% FBS-conditioned media that were separately treated with 5 and 30 µM oxaliplatin for 72 h. Also, P and FUR cells cultured in 1% FBS-conditioned medium were daily treated with 15 and 40 µM 5-FU for 72 h and collected cell culture supernatants to the isolation of EXO-P15, EXO-P40, EXO-FUR15, and EXO-FUR40. Treatments were conducted at low doses similar to the clinical doses (5 µM oxaliplatin and 15 µM 5-FU) and the resistance doses (30 µM oxaliplatin and 40 µM 5-FU), during which resistant cells continue to survive and proliferate. All collected conditioned media were centrifuged to remove dead cells and stored at -80^0^ C before exosome purification.

### Exosome isolation

Exosomes were purified from collected conditioned media (CCM) by sequential centrifugation at 4^0^C. CCM was centrifuged sequentially for 20 min at 500 x g to remove cell contamination, 25 min at 2500 x g to eliminate debris and apoptotic bodies, and 45 min at 14,600 x g to remove microvesicles. The cell supernatants were concentrated and purified with Ultra Amicon 100 KD at 4000 x g. Finally, the exosomes were isolated at 100,000 × g for 70 min by ultracentrifugation. Exosome pellets were dissolved in PBS plus 2.5 mM HEPES and stored at -80^0^C before experiments. The protein concentration of the exosomes was measured using a BCA assay kit (DNAbioTech, Iran).

### Exosome characterization

Transmission electron microscopy (TEM) and scanning electron microscopy (FSEM) were used to determine the size and morphology of exosomes. Briefly, 20 µL of exosome solution was dropped onto formvar carbon film coated on a 300 mesh copper grid (EMS) for 2 min. After removing excess liquid with filter paper, 20 µL of 2% aqueous uranyl acetate was used for staining negatively for 1–2 min and then allowed to dry at room temperature. Finally, grids were observed under a transmission electron microscope (Zeiss, EM10C) with an accelerated voltage of 100 kV. For FSEM, exosomes were dehydrated by air drying, coated with gold metal, and scanned at 50000x magnification using electron microscopy. DLS (Malvern Instruments) was performed to analyze the diameter and membrane electrical charge (zeta potential). Also, exosomes are recognized by their protein hallmarks, such as tetraspanins and heat shock proteins (Kalluri 2016). We characterized CD9 and Hsp70 as exosomal marker proteins by western blot. Exosomes from parental (EXO-P), oxaliplatin-resistant (EXO-OXR), or 5-FU-resistant (EXO-FUR) cells were employed to treat cells.

### Cell viability/proliferation assay

We performed the MTT assay to estimate cell viability and proliferation. Cells were cultured in 96-well plates, incubated at 37^0^C under 5% CO2 for 24 h, then treated with oxaliplatin or 5-FU and, or exosomes. Cell culture supernatants were removed after 72 h treatment, and 20 µl of MTT solution (5 mg/ml) was added, then incubated at 37^0^ C and 5% CO2 for 3–4 h. Finally, 100 µL of DMSO was added, and the optical density of each well was read at 570 nm by an ELISA reader. The data was analyzed using Graph Pad Prism. Relative resistance (RR) was calculated as IC_50_ of resistance cell/ IC_50_ of parental cell and reported.

### Wound healing assay

Cells were culture at 8 × 10 ^4^ cells in 12-well plates with a complete medium containing 10% FBS to reach 80% confluency of monolayer cells. The cells were washed twice with PBS to remove the FBS effect, and incubated in a serum starvation medium containing 0.5% FBS overnight. The next day, a straight scratch was generated in the middle of each plate well with a sterile 100 µl pipette tip and washed off detached cells twice with PBS. Then, parental cells were treated with 1 µg/ml of different exosomes per 1500 cells in fresh culture medium containing 0.5% FBS, and untreated OXR, FUR, and P cells remained as controls. Images of the scratched areas were captured at 0 and 24 h across 4 or 5 distinct fields using an inverted light microscope. The migration rates of cells were estimated by measuring the closure of two scratched edges using ImageJ software 1.52v (National Institutes of Health, USA).

### Cell invasion assay

The invasion experiment was performed using transwell culture inserts with 8.0-µm pores. 45 µL of 1:5 RPMI1640-diluted matrigel was added to the upper filter insert of wells and incubated at 37^0^ C for 6 h. Unattached cells were washed two times with 1% FBS medium and 3.5 × 10^4^ cells per well with 100 µg/ml exosomes in 100 µl of 1% FBS medium were seeded in the upper chamber. 600 µL of 20% FBS medium was also added as a chemoattractant to the lower chamber of the insert. The cells were allowed to invade at 37^0^ C, 5% CO2 incubator for 48 h. Then, the remaining cells on the upper surface of the filter inserts were removed gently using cotton swabs. Invaded cells to the lower surface of the filter inserts were fixed with methanol at 4^0^ C for 20 min and stained with 1% crystal violet solution for 30 min. Images were captured using an Olympus BX40 microscope and analyzed by ImageJ software 1.52v (National Institutes of Health, USA).

### Western blot analysis

We lysed the cells and exosomes using a lysis buffer and then performed Western Blotting as described previously (Shadboorestan et al. 2019). In brief, lysates were separated on sodium dodecyl sulfate-polyacrylamide gel electrophoresis (10%) and transferred to a PVDF membrane (Roche, Mannheim Germany). The membranes were blocked with 5% skim milk dissolved in tris-buffered saline containing Tween‐20 (TBST) for 90 min at room temperature and incubated with primary antibodies overnight at 4 °C. Then, the blots were washed with TBST buffer and incubated with Anti-rabbit/or anti-mouse IgG horseradish peroxidase (HRP)-conjugated secondary antibody (1:5000; BioRad, Hercules, CA, USA) for 1 h at room temperature. Finally, we detected the protein bands using the BM chemiluminescence detection system (Roche) and quantified them with Image J software 1.52v (National Institutes of Health, USA). The primary antibodies used for western blot were as follows: anti-ITGβ1 (1:1000, Cell Signaling; 3497), anti-ITGβ4 (1:1000, Cell Signaling; 4707), anti-ITGαv (1:1000, Cell Signaling; 6089), anti-ITGβ3 (1:1000, Cell Signaling; 4702), anti-CD9 (1:750, Biolegend; 312102), anti-β-actin (1:1000, Santa Cruz Biotech; 47778), anti-HSP70 (1:500, Cell Signaling; 4872), and anti-mmp-9 (1:500, Cell Signaling; 3852).

### Cell cycle and apoptosis analysis by flow cytometry

P, OXR, and FUR cells were cultured in 12 well plates. After 24 h, P cells were treated with 1 µg/ml exosomes per 500 cells. 72 h after treatment, all cells were trypsinized and centrifuged at 1500 rpm for 5 min. Cell pellets were suspended in PBS for flow cytometry analysis as previously described (Aliebrahimi et al. 2018). For cell cycle analysis, 70% cold ethanol was added to cell suspensions for 2 h at 4^0^ C to fix the cells. The solutions were centrifuged and the obtained pellets were stained with propidium iodide (PI) solution in the dark. Then, the solutions were incubated at room temperature for 30 min. DNA content analysis was performed using a FACS Calibur flow cytometer (BD Biosciences, San Jose, CA, USA). To investigate apoptosis by Annexin V/PI staining, 100 µl binding buffer was added to cell pellets and incubated with 5 µl Annexin V-FITC antibody (Invitrogen, USA) and 10 µl of PI (2 µg/ml) at room temperature for 15 min. Finally, 400 µl of binding buffer was added, and cell count was analyzed by flow cytometry.

### Statistical analysis

Statistical analyses were performed using GraphPad Prism version 8.4.3 software (GraphPad Software, USA). One-way ANOVA, followed by Dunnett’s and Tukey’s multiple comparisons tests, was used for analyses involving three or more groups. Nonlinear regression was utilized to determine IC_50_ values. *P <* 0.05 was considered significant.

## Results

### Characterization of exosomes

**Fig. 1:**
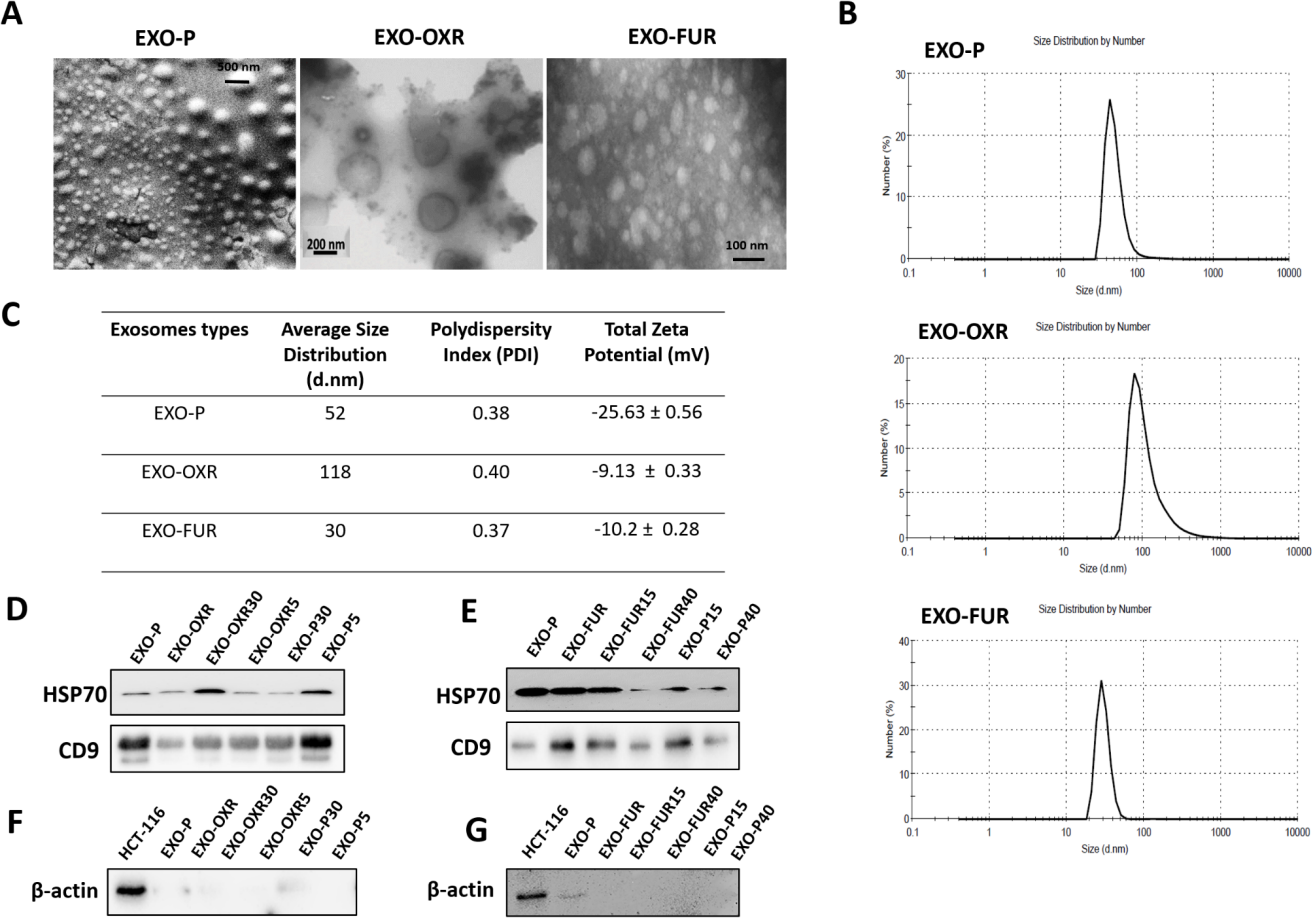
Characterization of exosomes. **A** Representative of EXO-P with a scanning electron microscope that scanned at 50000x magnification. EXO-OXR and EXO-FUR detected by TEM. **B** Representative size distribution graphs of DLS-based detection. **C** The table represents exosome average size and zeta potential measured in Zetasizer. **D**- **E** WB results show HSP70 and CD9 proteins for exosomal markers. **F-G** β-actin expression in exosomes was detected by western blot.

Exosomes were isolated using ultracentrifugation to examine the effects of different resistant exosomes on the behavior of parental cells. We obtained exosomes from oxaliplatin-resistant (OXR) cells and 5-FU-resistant (FUR) cells, as well as from oxaliplatin-treated OXR cells, 5-FU-treated FUR cells, parental (P) cells, and drug-treated parental cells. Subsequently, we characterized the exosomes. Morphology and size were evaluated using FSEM and TEM (Fig. 1A, Supplementary Fig. S1A). The diameter and membrane electrical charge of exosomes were confirmed through DLS. The parental, OXR, and FUR exosome sizes differed, measuring 30 nm for EXO-FUR, 52 nm for EXO-P, and 118 nm for EXO-OXR (Fig. 1B-C, Supplementary Fig. S1B). Western blot analysis confirmed the presence of exosome markers CD9 and HSP70 (Fig. 1D-E), while β-actin was not detected in any of the exosomes (Fig. 1F-G).

### OXR and FUR exosomes promote chemoresistance in parental cells

**Fig. 2:**
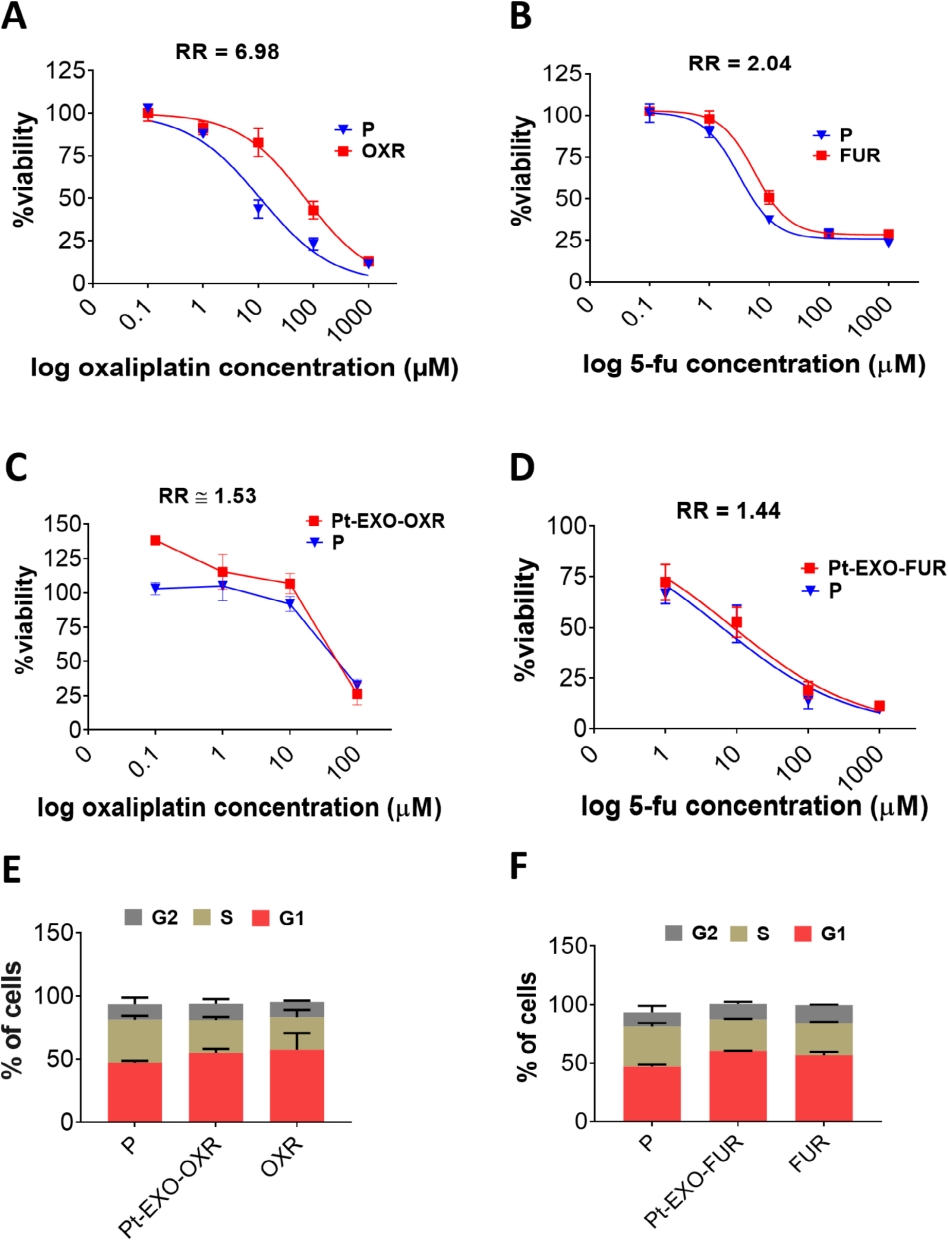
Exosomes from oxaliplatin- and 5-FU-resistant cells develop chemoresistance in parental cells. **A-B** OXR, FUR, and P cells were treated with different concentrations of oxaliplatin (**A**) and 5-FU (**B**) for 72 h. Cell viability was measured using the MTT assay. The curves represent the RR (relative resistance) of oxaliplatin and 5-FU resistant cells. **C**-** D** parental cells were treated with 1 µg/ml of EXO-OXR or EXO-FUR / 500 cells for 72 h. Untreated and exosome-treated P cells were treated with different concentrations of oxaliplatin or 5-FU for 72 h and the MTT assays were performed. The RR of exosome-treated vs. untreated parental cells was calculated. **E**-** F** Representative the effects of oxaliplatin-resistant cell exosomes(EXO-OXR) (**E**) and 5-FU-resistant cell exosomes (EXO-FUR) (**F**) on the cell cycle of parental cells (P) by flow cytometry. The error bars were obtained from triplicate samples. Data are mean ± SD.

Given that exosomes derived from chemoresistant cells modify chemosensitive cell phenotypes (Milman et al. 2019; Li et al. 2020), we explored whether resistant cell exosomes could transfer oxaliplatin and 5-FU resistance to recipient cells through increased IC_50_. The Evaluation of the IC_50_ for parental (P), oxaliplatin-resistant (OXR), and 5-FU-resistant (FUR) cells after 72 h treatment with oxaliplatin and 5-FU revealed that OXR cells exhibited a 6.98-fold and FUR cells a 2-fold increase in resistance compared to parental cells (Fig. 2A-B). The specific IC_50_ values for each cell type are detailed in Table 1. To examine the effect of exosomes from OXR (EXO-OXR) and FUR (EXO-FUR) on the changes of IC_50_ of oxaliplatin and 5-FU in parental cells, we treated parental cells with EXO-OXR or EXO-FUR for 72 h. Subsequently, we exposed both exosome-treated and untreated P cells to various concentrations of oxaliplatin and 5-FU. The parental cells treated with OXR and FUR exosomes indicated a significantly higher IC_50_ than untreated ones. IC_50_s of parental cells treated with EXO-OXR and EXO-FUR were increased to 90.71 ± 3.3 µM (vs. 59.10 ± 0.05) and 8.93 ± 0.092µM (vs. 6.18 ± 0.11), respectively. The relative resistance (RR) of oxaliplatin in EXO-OXR-treated parental cells was approximately 1.5 times greater than that of untreated parental cells (Fig. 2C). The RR of 5-FU for EXO-FUR-treated parental cells was 1.44 times higher than that of exosome-untreated parental cells (Fig. 2D). We next performed flow cytometry to analyze the cell cycle. The parental cells treated with EXO-OXR and EXO-FUR exhibited cell cycle patterns similar to those of the OXR and FUR cells. P cells showed a smaller G1 phase and a larger S phase than FUR and OXR cells. EXO-OXR- and EXO-FUR-treated parental cells, like OXR and FUR cells, displayed longer G1 phases and shorter S phases (Fig. 2E-F, Supplementary Fig. S2), indicating the development of oxaliplatin and 5-FU resistance through exosomes.

**Table 1:** IC_50_ of oxaliplatin and 5-FU in parental and resistant cells after 72 h

Cell lines	IC_50_ (µM)	
	Oxaliplatin	5-FU
Parental Cells (P)	9.35 ± 1.77	13.6 ± 0.19
Oxaliplatin Resistant Cells (OXR)	65.28 ± 5.89	-
5-FU Resistant Cells (FUR)	-	27.52 ± 0.06

### Parental cells treated with different OXR exosomes show distinct resistance behaviors, although all exhibit high ITGβ3 and low ITGβ4 patterns

**Fig. 3:**
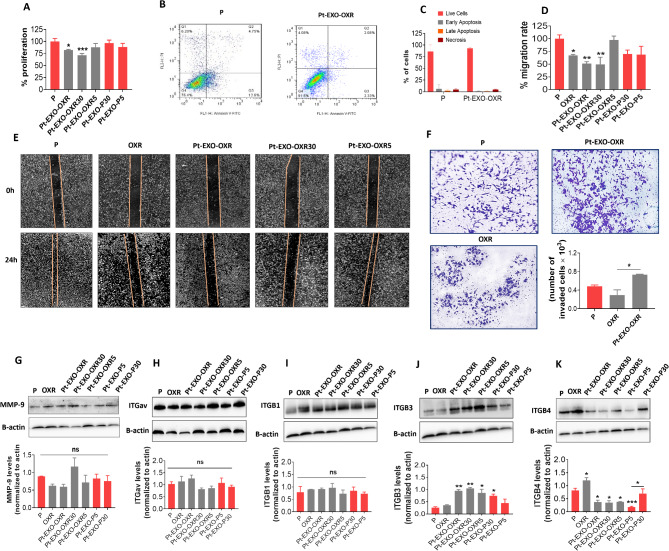
Effects of exosomes from untreated and oxaliplatin-treated OXR or parental cells on parental cell behaviors. **A** shows the impact of exosomes on parental cell proliferation. 4000 parental cells were cultured in 96-well plates. After 24 h, the cells were treated with different groups of untreated or oxaliplatin-treated OXR or P cell exosomes with a concentration of 1 µg/ml EXO/ 500 cells. 72 h after exosome treatment was assessed by MTT assay. **B** shows the effects of oxaliplatin-resistant exosomes (EXO-OXR) on the apoptosis of parental cells by flow cytometry. **C** Quantification of data from **B**. **D** Quantification of data from **E. E** Representative images of scratch closure rate after 24 h in migrated cells, including parental cells (P), oxaliplatin-resistant (OXR) cells, and parental cells that were treated with different types of exosomes derived from untreated OXR cells or oxaliplatin-treated OXR cells. **F** Representative images of crystal violet staining of invaded P, OXR, and oxaliplatin-resistant exosomes-treated P cells (Pt-EXO-OXR) from transwell culture inserts coated with matrigel and quantification of invaded cell images. Data are mean ± SD. **G** Western blot images and their quantification of MMP-9 from P, OXR cells, and P cells treated with OXR exosomes or oxaliplatin-treated resistant or parental cell exosomes. **H-K** Representative ITG levels in parental cells after treatment with exosomes derived from OXR cells and oxaliplatin-resistant and parental cells treated with 5 or 30 µM oxaliplatin. Data are mean ± s.e.m. *P < 0.05, **P < 0.01, ***P < 0.001. ns, non-significant.

Next, we investigated the impact of exosomes derived from oxaliplatin-resistant and oxaliplatin-treated cells on cell behaviors. We observed that the proliferation rate of parental cells was inhibited after treatment with exosomes from OXR cells (EXO-OXR) or exosomes from OXR cells treated with 30 µM oxaliplatin (EXO-OXR30). In contrast, treatment with exosomes from OXR cells treated with 5 µM oxaliplatin (EXO-OXR5) did not inhibit the proliferation of the parental cells. Also, exosomes secreted from oxaliplatin-treated parental cells (EXO-P5 and EXO-P30) did not have any effect on the proliferation rate of parental cells (Fig. 3A). We observed that treating parental cells with different concentrations of parental exosomes (EXO-P) did not significantly affect cell viability or proliferation rates; however, high doses of exosomes (50 µg/ml) resulted in reduced viability (Supplementary Fig. S3). We investigated apoptosis using flow cytometry to confirm that the reduced proliferation was not due to exosome toxicity. Parental cells treated with EXO-OXR showed no apoptotic effects compared to untreated parental cells (Fig. 3B-C). The cell cycle results also confirm reduced proliferation due to mimicking the resistant cell cycle. (Fig. 2E). Next, we examined the effects of exosomes on cell migration and invasion. We found that OXR cells exhibited reduced migration compared to the parental cells. When parental cells were treated with either EXO-OXR or EXO-OXR30, their migration was suppressed, indicating a therapeutic failure at 30 µM oxaliplatin. While EXO-OXR5 did not affect migration (Fig. 3D-E) or proliferation of parental cells (Fig. 3A), suggesting a potential therapeutic effect for 5 µM oxaliplatin. OXR cells did not display any invasion traits, and the treatment of parental cells with EXO-OXR had a negligible impact on their invasion (Fig. 3F). Considering the role of matrix metalloproteinase (MMP) proteins in the invasion, we also examined the levels of MMP-9 protein. The western blot showed no significant differences in MMP-9 levels between OXR cells and EXO-OXR-treated parental cells compared to the parental cells alone. Furthermore, MMP-9 levels in the parental cells remained unchanged after treatment with exosomes from either oxaliplatin-treated resistant cells or parental cells, indicating that this protein was not affected by oxaliplatin resistance or treatment (Fig. 3G). These results indicate that oxaliplatin resistance is not associated with invasive traits, such as increased migration and invasion.

Given the significance of integrins in cellular behaviors (Hamidi and Ivaska 2018), we examined integrin expressions in cells treated with exosomes. Parental cells treated with exosomes from untreated OXR cells or oxaliplatin-treated OXR and P cells showed increased ITGβ3 and decreased ITGβ4 levels. However, ITGβ4 levels remained unchanged after EXO-P30 treatment, and ITGβ3 levels remained unchanged after EXO-P5 treatment (Fig. 3H-K). These findings indicate that parental cells treated with EXO-OXR, EXO-OXR30, or EXO-OXR5 displayed similar integrin patterns. However, EXO-OXR and EXO-OXR30 reduced the migration and proliferation of parental cells, while EXO-OXR5 did not exhibit these effects. This suggests that exosome integrins may determine cellular behaviors. Furthermore, parental cells treated with EXO-P30 exhibited an increase in ITGβ3 expression, while those treated with EXO-P5 demonstrated a decrease in ITGβ4 expression (Fig. 3J-K). However, these changes did not impact cell migration, proliferation, or MMP-9 levels (Fig. 3A, D, G, Supplementary Fig. S4). These findings highlight the significance of increased ITGβ3 with reduced ITGβ4 in developing resistance to oxaliplatin and may also facilitate the transfer of therapeutic effects via exosomes.

### High levels of exosomal ITGαv and ITGβ3 are associated with oxaliplatin resistance, while lower exosomal ITGβ3 levels suggest a more favorable prognosis at the secondary sites

**Fig. 4:**
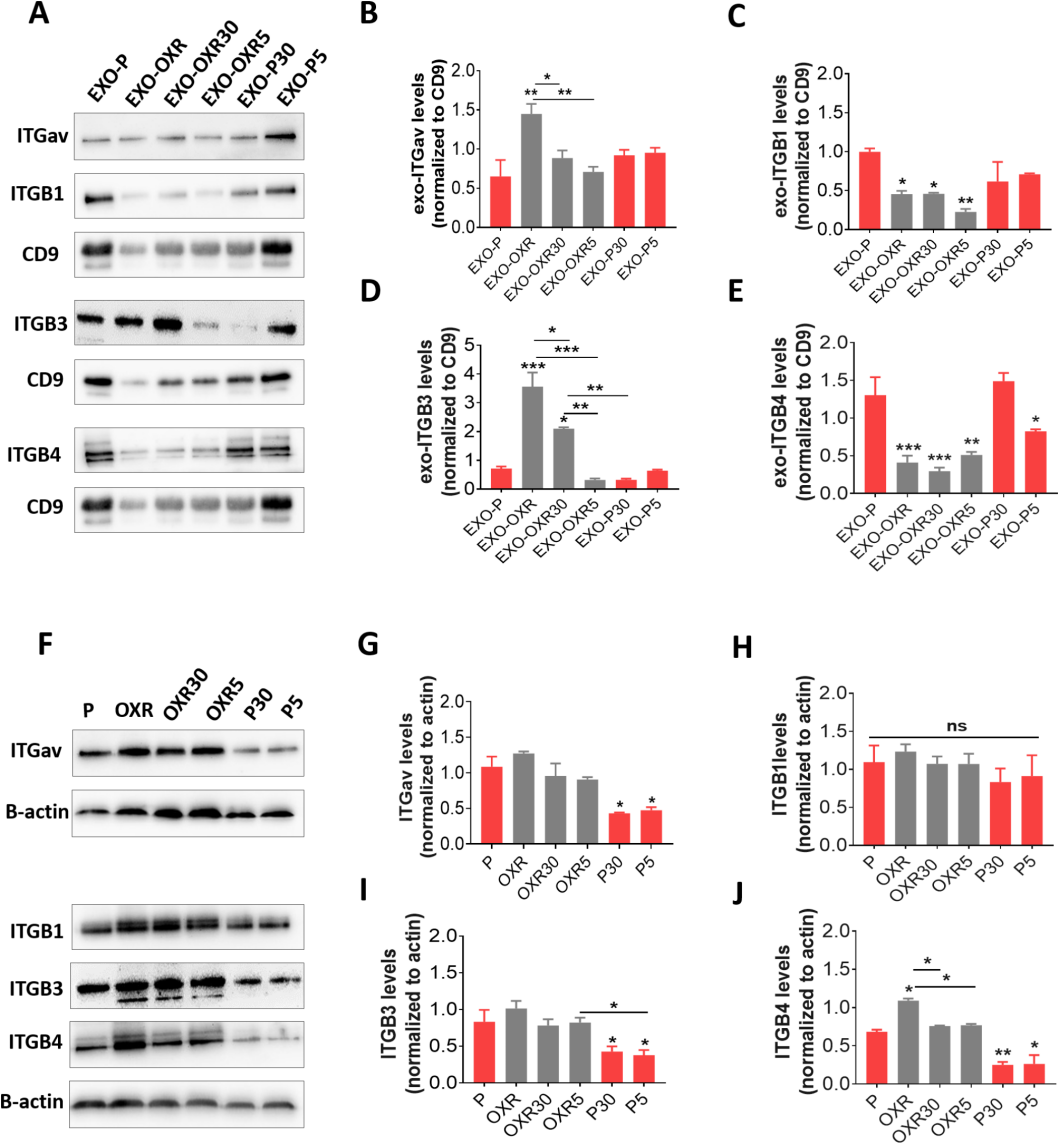
Integrin expression patterns in oxaliplatin resistance and treatment at exosomal and cellular levels. **A** Representative of western blot images in exosomal ITG levels of parental (P) and oxaliplatin-resistant (OXR) cells after treatment with 5 or 30 µM oxaliplatin and untreated cells. **B-E** Quantifying data from **A**. **F** Western blot images in their cellular ITG levels. **G-J** Quantification of data from **F**. Data are mean ± s.e.m. *P < 0.05, **P < 0.01, ***P < 0.001. ns, non-significant.

We examined whether different groups of exosomes that induce similar integrin patterns but elicit varying biological behaviors have distinct integrin expressions. Additionally, we compared these patterns with those of their originating cells. The western blot results showed that EXO-OXR had higher ITGαv and ITGβ3 levels but lower ITGβ4 and ITGβ1 levels than EXO-P (Fig. 4A-E). In contrast to exosomes, OXR cells only showed higher ITGβ4 levels (Fig. 4F-J). After oxaliplatin treatment, the elevated integrin levels in OXR cells and OXR exosomes were reduced. The levels of ITGαv and ITGβ3 were diminished in EXO-OXR5 and EXO-OXR30, while ITGβ4 levels decreased in both OXR5 and OXR30 cells. In contrast, oxaliplatin treatment caused a reduction in ITGαv, ITGβ3, and ITGβ4 in parental cells (Fig. 4F-G, I-J) and a decrease of ITGβ4 in parental cell exosomes at 5µM (Fig. 4A, E). No significant difference was observed in the effectiveness of 5 µM and 30 µM oxaliplatin on cell integrins (Fig. 4F-J). However, 5 µM oxaliplatin caused a more significant reduction of ITGβ3 in resistant exosomes than 30 µM oxaliplatin. EXO-OXR5 displayed similar ITGβ3 levels to EXO-P, while EXO-OXR30 demonstrated increased levels compared to parental exosomes (Fig. 4A, D). This difference was particularly evident at the secondary site, where EXO-OXR5 did not transfer resistant features to parental cells, in contrast to EXO-OXR and EXO-OXR30 (see Fig. 3A, D-E).

### Parental cells treated with various FUR exosomes show varying degrees of invasive behaviors, but all display increased ITGαv and decreased ITGβ4 levels

**Fig. 5:**
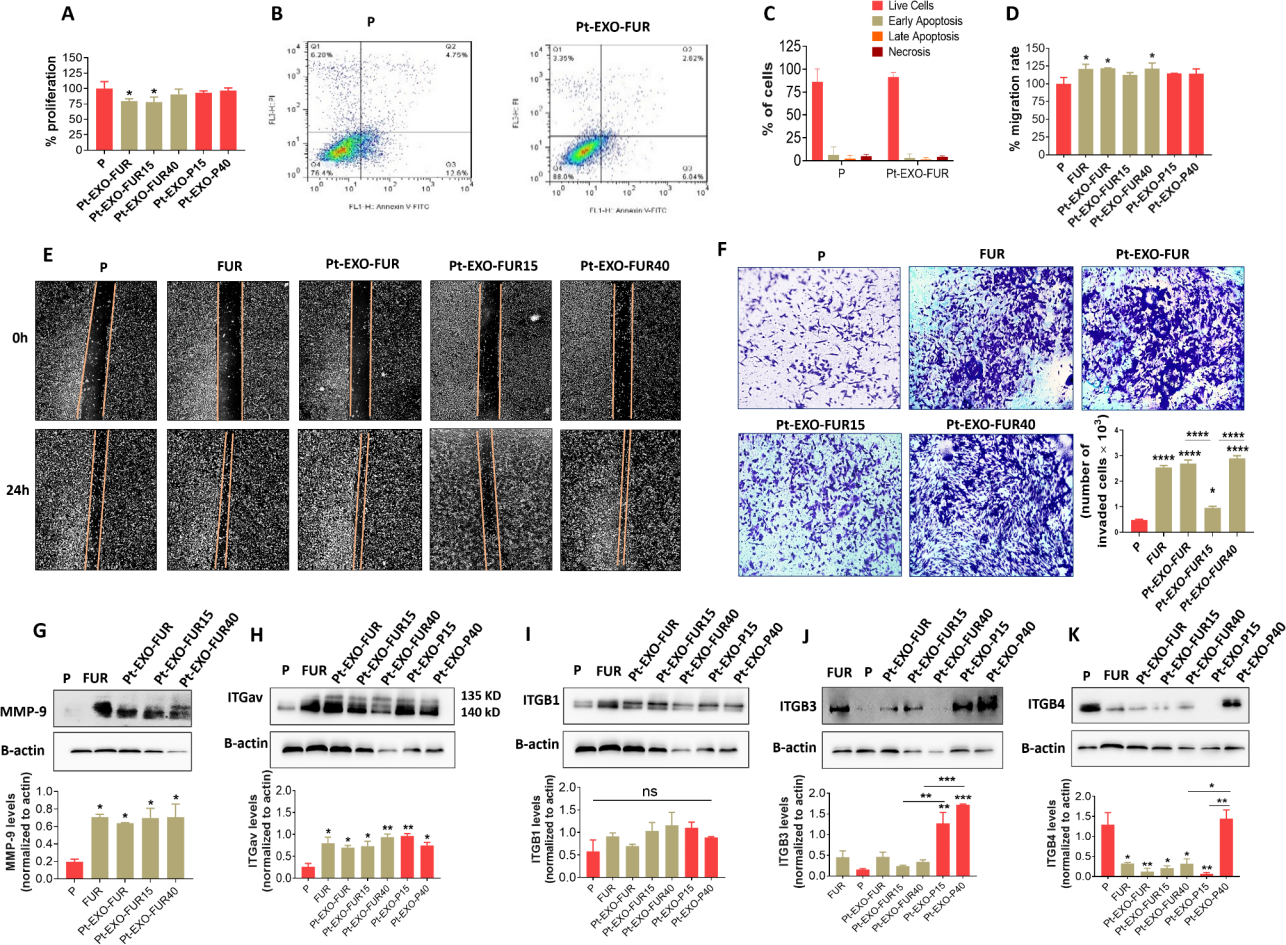
Effects of exosomes from untreated and 5-FU-treated FUR or parental cells on parental cell behaviors. **A** shows the impact of exosomes on parental cell proliferation. 4000 parental cells were cultured in 96-well plates. After 24 h, the cells were treated with different groups of untreated or 5-FU-treated FUR or P cell exosomes with a concentration of 1 µg/ml of exosomes per 500 cells. 72 h after exosome treatment, cell viability was assessed by MTT assay. **B** shows the effects of 5-FU-resistant exosomes (EXO-FUR) on the apoptosis of parental cells by flow cytometry. **C** Quantification of data from **B**. **D** Quantification of data from **E**. **E** Representative images of scratch closure rate after 24 h in migrated cells including parental cells (P), 5-FU-resistant (FUR) cells, and parental cells that were treated with different types of exosomes derived from untreated FUR cells or 5-FU-treated FUR. **F** Representative images of crystal violet staining of invaded P, FUR, 5-FU-resistant exosomes-treated P cells (Pt-EXO-FUR), and parental cells treated with 5-FU-treated FUR exosomes (Pt-EXO-FUR15 and Pt-EXO-FUR40) from transwell culture inserts coated with matrigel and quantification of invaded cell images. Data are mean ± SD. **G** Western blot images and their quantification of MMP-9 from P, FUR cells, and P cells treated with 5-FU-treated FUR cell exosomes (EXO-FUR15 and EXO-FUR40). **H-K** Representative ITG levels in parental cells after treatment with exosomes derived from FUR cells and FUR and parental cells treated with 15 or 40 µM 5-FU. Data are mean ± s.e.m. *P < 0.05, **P < 0.01, ***P < 0.001, ****P < 0.0001. ns, non-significant.

**Fig. 6:**
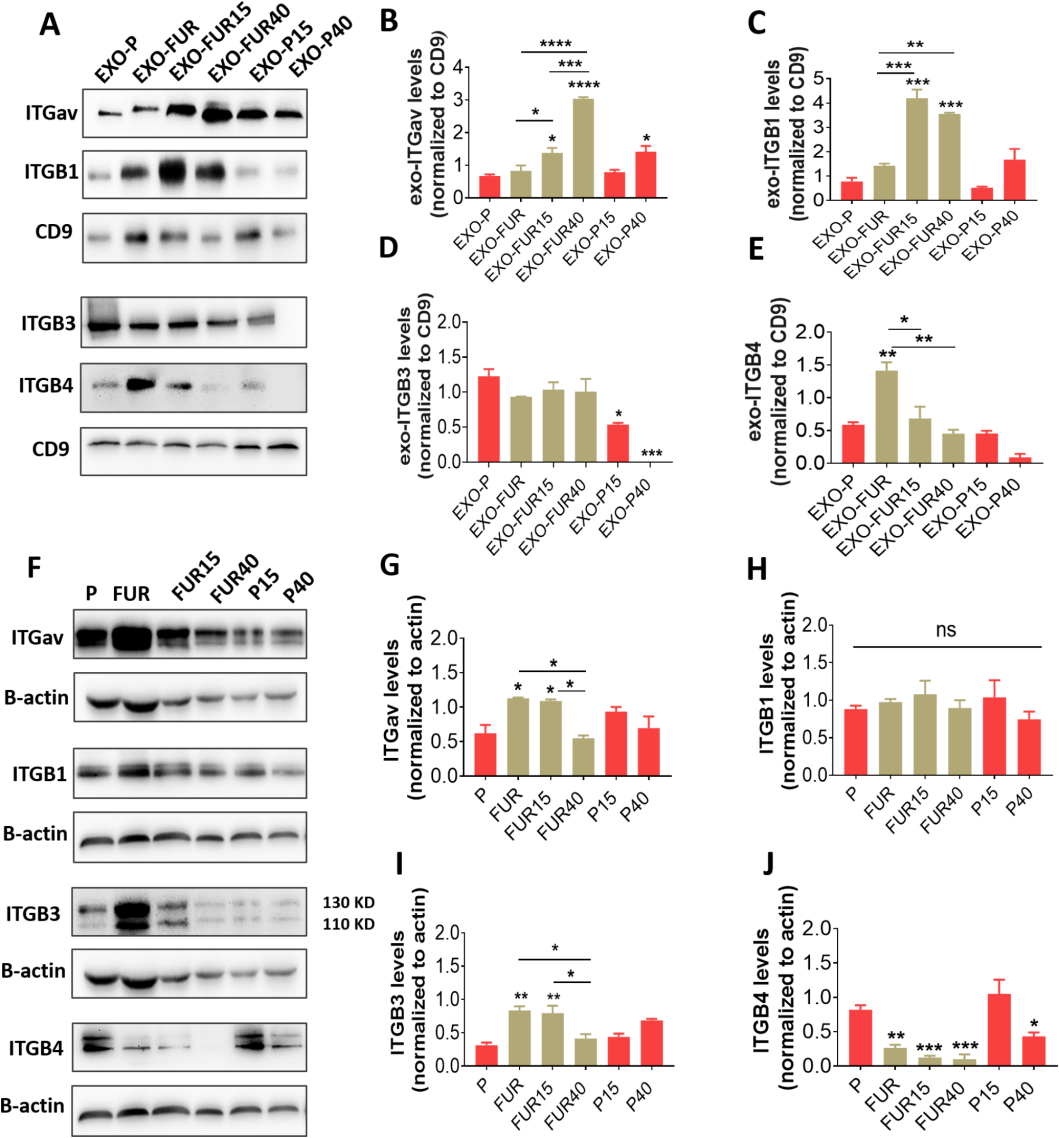
Integrin expression patterns in 5-FU resistance and treatment at exosomal and cellular levels. **A-E** Data display Western blot images (**A**) and quantification of integrin expressions in exosomes of parental (P) and 5-FU resistant cells (FUR) after treatment with 15 or 40 µM 5-FU, as well as untreated cells (**B-E**). **F-J** Western blot images (**F**) and quantification of integrin expressions in their cell groups (**G-J**). Data are mean ± s.e.m.*P < 0.05, **P < 0.01, ***P < 0.001, ****P < 0.0001. ns, non-significant.

To further investigate, we examined the effects of exosomes from 5-FU-resistant and 5-FU-treated cells on the behaviors of secondary cells. We observed that parental cells exhibited reduced proliferation after treatment with exosomes from FUR cells (EXO-FUR) or exosomes from FUR cells treated with 15 µM 5-FU (EXO-FUR15), similar to those treated with OXR exosomes (Fig. 3). In contrast, treating parental cells with exosomes from FUR cells treated with 40 µM 5-FU (EXO-FUR40) or those secreted from 5-FU-treated parental cells (EXO-P15 and EXO-P40) did not result in any inhibitory effects (Fig. 5A). Additionally, Parental cells treated with EXO-FUR showed no apoptotic effects compared to untreated cells (Fig. 5B-C), indicating that the observed reduction in viability was not due to exosome toxicity. This was further confirmed by the cell cycle results (Fig. 2F). We next investigated the impact of exosomes on cell migration and invasion. The FUR cells demonstrated increased migration compared to the parental cells. EXO-FUR and EXO-FUR40 also enhanced migration in parental cells, while EXO-FUR15 did not show this effect (Fig. 5D-E). Parental cells treated with exosomes from 5-FU-treated parental cells (EXO-P15 or EXO-P40) exhibited no significant changes in migration (Fig. 5D, Supplementary Fig. S4). The invasion results showed a 5-fold increase in invasion of FUR cells compared to the parental cells. EXO-FUR, EXO-FUR15, and EXO-FUR40 also enhanced the invasion of parental cells. Interestingly, parental cells treated with EXO-FUR15 exhibited a more than 2.5-fold reduction in invasion compared to those treated with EXO-FUR or EXO-FUR40 (Fig. 5F). The observed increased invasion in FUR cells and parental cells treated with various EXO-FUR groups was linked to higher levels of MMP-9 protein (Fig. 5G). Furthermore, we observed that when parental cells were treated with exosomes from FUR cells or exosomes derived from 5-FU-treated FUR or parental cells, the level of ITGαv increased, and ITGβ4 decreased, except ITGβ4 after treatment with EXO-P40. Moreover, in the various exosome treatments, the levels of ITGβ1 and ITGβ3 remained unchanged. However, there was a significant increase in ITGβ3 levels following treatment with exosomes isolated from 5-FU-treated parental cells (Pt-EXO-P15 or Pt-EXO-P40) (Fig. 5H-K), indicating that 5-FU treatment affects the expression of ITGβ3 in both exosomes and the recipient cells (Figs. 5J and 6D). These results demonstrated that parental cells treated with various exosomes (EXO-FUR, EXO-FUR15, or EXO-FUR40), exhibited similar integrin patterns (high ITGαv and low ITGβ4), despite differences in their behaviors. EXO-FUR or EXO-FUR40 enhanced migration and invasion, whereas EXO-FUR15 did not promote migration and reduced invasion compared to EXO-FUR or EXO-FUR40. Parental cells treated with EXO-FUR or EXO-FUR15 showed inhibited proliferation, while those treated with EXO-FUR40 did not show such effects. Data suggest that integrin expression patterns in exosomes may affect the behaviors of recipient cells, as observed in oxaliplatin resistance (Fig. 3).

### 5-FU resistance increases exosomal ITGβ4, while 5-FU treatment increases ITGαv; lowering these levels improves therapeutic outcomes at secondary sites

Next, we investigated the differences in integrin patterns of exosomes derived from 5-FU resistant and treated cells and compared these patterns to the integrin expression in the cells themselves. Western blot analysis demonstrated that in FUR exosomes (EXO-FUR), ITGβ4 was the only integrin that exhibited increased expression levels compared to those found in parental cell exosomes (EXO-P) (Fig. 6A-E). In contrast, FUR cells displayed higher expression levels of ITGαv and ITGβ3 than the parental cells; however, they showed a decrease in ITGβ4 levels compared to the exosome levels (Fig. 6F-J). 5-FU treatment reduced the elevated levels of resistant integrins, specifically ITGαv and ITGβ3 in FUR cells at 40 µM (Fig. 6F-G, I), and reduced ITGβ4 in FUR exosomes at both 15 and 40 µM (Fig. 6A, E). Furthermore, 5-FU treatment increased the expression of ITGαv and ITGβ1 in FUR exosomes (EXO-FUR15 and EXO-FUR40). The EXO-FUR40 exhibited a twofold increase in ITGαv levels compared to EXO-FUR15 (Fig. 6A-C). 15 µM 5-FU did not affect integrin expressions in either FUR or parental cells (Fig. 6F-J). In contrast, 40 µM 5-FU decreased various integrins in parental cells (ITGβ4) and FUR cells (ITGαv and ITGβ3) (Fig. 6F-G, I-J). Parental cells treated with 5-FU showed reduced ITGβ3 levels at both doses and elevated ITGαv levels at 40µM in their exosomes (Fig. 6A-E). These results indicate that cells overexpressing ITGαv and ITGβ3 are resistant to 5-FU. Treatment with 5-FU reduced these overexpression levels in resistant cells while also increasing the levels of ITGαv in exosomes from both parental and resistant cells. This suggests that resistance resulting from 5-FU treatment may be transferred through exosomes. A reduction in ITGβ4 and ITGαv levels in resistant exosomes indicates a positive therapeutic outcome at the secondary sites (see Fig. 5).

## Discussion

As the incidence of CRC rises, the number of patients with advanced metastatic CRC (mCRC) is also increasing due to drug resistance and treatment failures (Siegel et al. 2014; Advani and Kopetz 2019). Integrins have been investigated as therapeutic targets for approximately forty years and have recently been used as probes in cancer imaging. However, their complexity and conflicting characteristics pose considerable challenges in developing effective integrin-based treatments (Pang et al. 2023; Notni et al. 2016; Feng et al. 2020). This study highlights the importance of exosome integrins compared to cell integrins in developing 5-FU and oxaliplatin resistance in CRC cells.

**Fig. 7:**
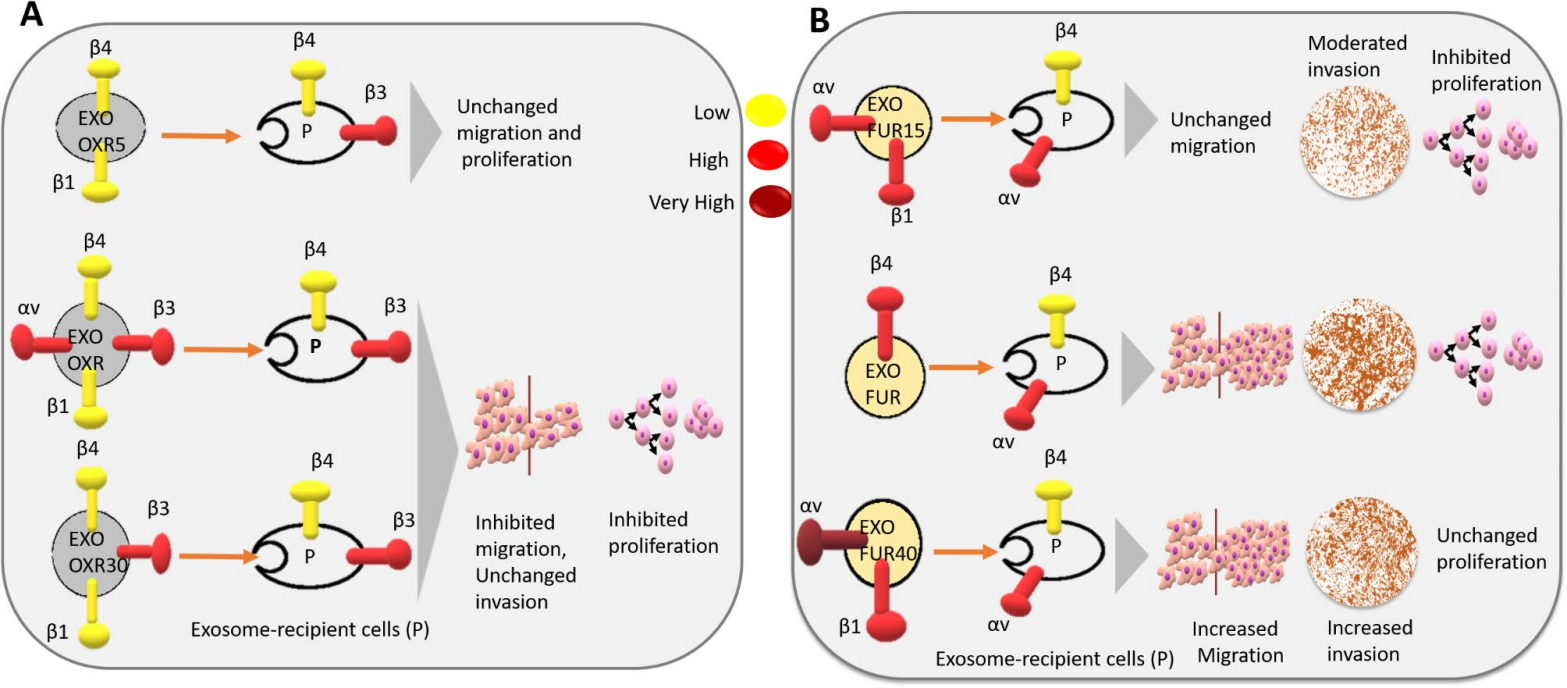
Exosome integrins determine resistant behaviors. **A** shows integrin expressions in oxaliplatin resistance and treatment (5 or 30 µM) at the levels of exosomes and exosome-recipient cells and the impact of exosomes on recipient parental cell behaviors (P). **B** shows integrin expressions in 5-FU resistance and treatment (15 or 40 µM) at the levels of exosomes and exosome-recipient cells and the impact of exosomes on recipient parental cell behaviors (P).

Our study demonstrated that OXR cells and OXR exosome-recipient cells exhibited decreased migration, while their invasion remained unchanged. The OXR cells displayed only high ITGβ4 levels, while EXO-OXR-recipient cells showed increased ITGβ3 and decreased ITGβ4 levels (Figs. 3 and 4). This indicates that two resistant cell types exhibited similar resistant behaviors while displaying distinct integrin patterns. Furthermore, The EXO-OXR, EXO-OXR5, and EXO-OXR30-recipient cells exhibited similar integrin patterns, characterized by high ITGβ3 and low ITGβ4 levels. However, these cells displayed different behaviors. EXO-OXR and EXO-OXR30 decreased migration and proliferation, while EXO-OXR5 did not elicit these effects (Figs. 3 and 7A). Likewise, Parental cells treated with EXO-FUR, EXO-FUR15, or EXO-FU40 displayed similar integrin expression profiles, characterized by increased ITGαv and decreased ITGβ4 levels. Nonetheless, they demonstrated varying degrees of invasive behaviors (Figs. 5 and 7B). These results demonstrate that distinct cellular integrin patterns can lead to similar cell behaviors, while the same integrin patterns may provide diverse cell behaviors. These findings indicate possible therapeutic failures associated with integrin targets, due to the heterogeneity observed in cell integrin expression and cellular behaviors (Cox 2021), emphasizing the intricacy of resistance mechanisms and thus reinforce the necessity of comprehending the interaction between exosomes and integrins in drug resistance.

ITGβ3 can develop drug resistance by activating prosurvival signaling pathways (Dolinschek et al. 2021) or inducing EMT phenotype (Wang et al. 2019). In this study, we observed that exosomes derived from oxaliplatin-resistant cells exhibited elevated levels of ITGβ3 (Fig. 4). These exosomes could impart resistance traits to the parental cells. A decrease in ITGβ3 levels in EXO-OXR was associated with an improved prognosis for reducing resistance behaviors at secondary sites. EXO-OXR5, which could not transfer oxaliplatin-resistant characteristics to recipient cells, exhibited lower ITGβ3 levels compared to both EXO-OXR and EXO-OXR30, which were capable of transferring resistance. (Figures 3 and 4). Consequently, oxaliplatin resistance was linked to elevated levels of exosomal ITGβ3, which conveyed non-invasive resistance behaviors.

Elevated ITGαv levels in cancer cells can induce enhanced migration and invasion (Li et al. 2013; WU et al. 2017; Ha et al. 2014) and facilitate cancer metastasis through exosomes (Krishn et al. 2019). Furthermore, the overexpression of ITGβ4 is associated with migratory and invasive behaviors, as well as reduced survival rates in various tumor cells, particularly in CRC patients (Beaulieu 2019; Draheim et al. 2010; Leng et al. 2016; Zhang et al. 2017). However, these characteristics may also be associated with lower levels of ITGβ4 expression (Mishra et al. 2015; Sordat et al. 1998; Rademaker et al. 2023). This study demonstrates that FUR exosomes only overexpressing ITGβ4 exhibit invasive characteristics of FUR cells (Figs. 5 and 6). FUR cells and EXO-FUR-recipient cells demonstrated enhanced migration and invasion behaviors. The 5-FU treatment increased ITGαv and decreased ITGβ4 in FUR cell exosomes (Fig. 6). The decrease of ITGβ4 in FUR cell exosomes after 5-FU treatment (EXO-FUR15 and EXO-FUR40) inhibited resistant migration and proliferation in parental cells. Furthermore, elevated ITGαv levels determine the invasion rate (Figs. 5 and 6). EXO-FUR40 exhibited more than a twofold increase in ITGαv compared to EXO-FUR15, resulting in more invasive behaviors in recipient cells than those observed with EXO-FUR15 (Figs. 5 and 6, and 7B). Indeed, a twofold reduction in ITGαv levels in EXO-FUR15 resulted in inhibited migration and a threefold decrease in invasion in recipient cells compared to those treated with EXO-FUR40.

Taken together, exosomes with high levels of ITGαv or ITGβ4 exhibited 5-FU resistance and invasive characteristics, while lower levels were associated with a more favorable prognosis of moderate invasive behaviors. Moreover, high exosomal ITGβ3 levels determine non-invasive oxaliplatin-resistant behaviors. Reducing this exosomal integrin level through oxaliplatin indicates desirable therapeutic effects by diminishing resistant behaviors at the secondary sites. Therefore, future research on exosomal integrins could provide promising targets for cancer diagnosis and assessing treatment response. Furthermore, no substantial change in cellular integrin reduction was found between 5 and 30 µM oxaliplatin. Oxaliplatin at 5 µM showed a larger therapeutic impact on secondary cells than 30 µM in EXO-OXR5-recipient cells (Figs. 3 and 4). In addition, 15 µM of 5-FU did not affect cell integrins (Fig. 5), but 40 µM showed a therapeutic effect by reducing resistant integrins. This contrasted with EXO-FUR40 (Figs. 5 and 6). These findings imply that exosomes are crucial to therapy outcomes, hence increasing dosage does not improve results, and highlighting the need to investigate exosome integrins in improving metastatic treatments (Vasan et al. 2019; Gatenby and Brown 2020; Wortzel et al. 2019). This study has some limitations. The analysis of decreased ITGβ1 in oxaliplatin-resistant exosomes and its increase in 5-FU-resistant exosomes was not conducted accurately, as the expression of this integrin remained unchanged in both the originating cells and the exosome-recipient cells. Moreover, further preclinical and clinical research studies will contribute to a more comprehensive and profound knowledge of the subject.

Finally, there are nearly 90 therapeutic drugs based on integrins. Nonetheless, only 7 drugs have successfully reached the market (Pang et al. 2023). A key lesson from the past illustrates that success relies on a profound comprehension of the functional mechanisms of integrins. Nonetheless, the roles of exosome integrins in metastasis (Hoshino et al. 2015) and drug resistance alter treatment approaches, highlighting their significance, as demonstrated in this study.

## Conclusion

Our study suggests that exosomal integrins, rather than the integrins on recipient cells, play crucial roles in determining resistance behaviors. High ITGβ3 exosomes and high ITGαv or ITGβ4 exosomes determine cell behaviors to oxaliplatin and 5-FU resistance. Decreasing their levels can indicate a favorable prognosis of therapy effects. Additionally, our findings reveal that while both the origin and recipient cells of the exosomes can create discrepancies between integrin expression patterns and cell behaviors, exosomal integrins are more reliable indicators of the behavioral characteristics of both the origin and recipient cells. Therefore, they serve as valuable targets for therapeutic and diagnostic purposes.

## Electronic supplementary material

Below is the link to the electronic supplementary material.

Supplementary Material 1

## Data Availability

No datasets were generated or analysed during the current study.

## Abbreviations

CRC: Colorectal Cancer
P: Parental cell (HCT-116)
OXR: Oxaliplatin-resistant cell
FUR: 5-FU-resistant cell
EXO: Exosome
P5 or P30: Parental cells treated with 5 or 30 µM oxaliplatin
OXR5 or OXR30: Oxaliplatin-resistant cells treated with 5 or 30 µM oxaliplatin
P15 or P40: Parental cells treated with 15 or 40 µM 5-FU
FUR15 or FUR40: 5-FU-resistant cells treated with 15 or 40 µM 5-FU
Pt-EXO: Parental cell treated with exosome
