# Supplementary material for: Exosomes and their distinct integrins transfer the characteristics of oxaliplatin- and 5-FU-resistant behaviors in colorectal cancer cells
Source: Mol Med. 2025 Feb 6;31:49. doi: 10.1186/s10020-025-01110-y (PMC11803997; doi:10.1186/s10020-025-01110-y)
Supplement: Supplementary file 1 — Supplementary Material 1 [file 10020_2025_1110_MOESM1_ESM.docx]

**
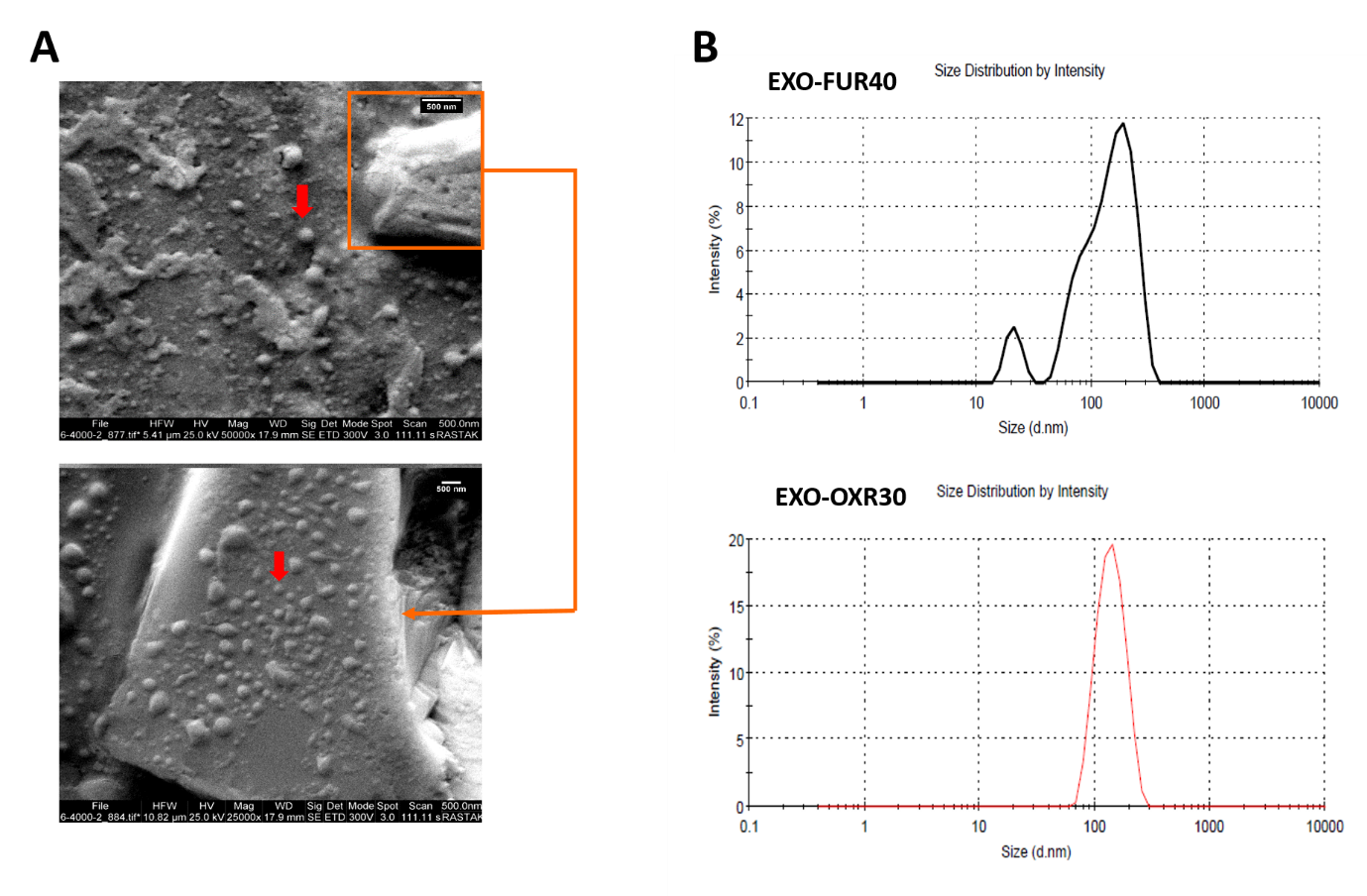
**

**Fig. S1** Characterization of exosomes. **A** Representative of exosome with a scanning electron microscope that scanned at 50000x magnification. The left image shows exosomes in the saline media of PBS plus HEPES. The right image represents exosomes on the salt crystal. Red arrows indicate exosomes. **B** Representative size distribution graphs of DLS-based detection for exosomes from 5-FU resistant cell treated with 40 μM 5-FU (EXO-FUR40) and exosomes from oxaliplatin resistant cell treated with 30 μM oxaliplatin (EXO-OXR30).


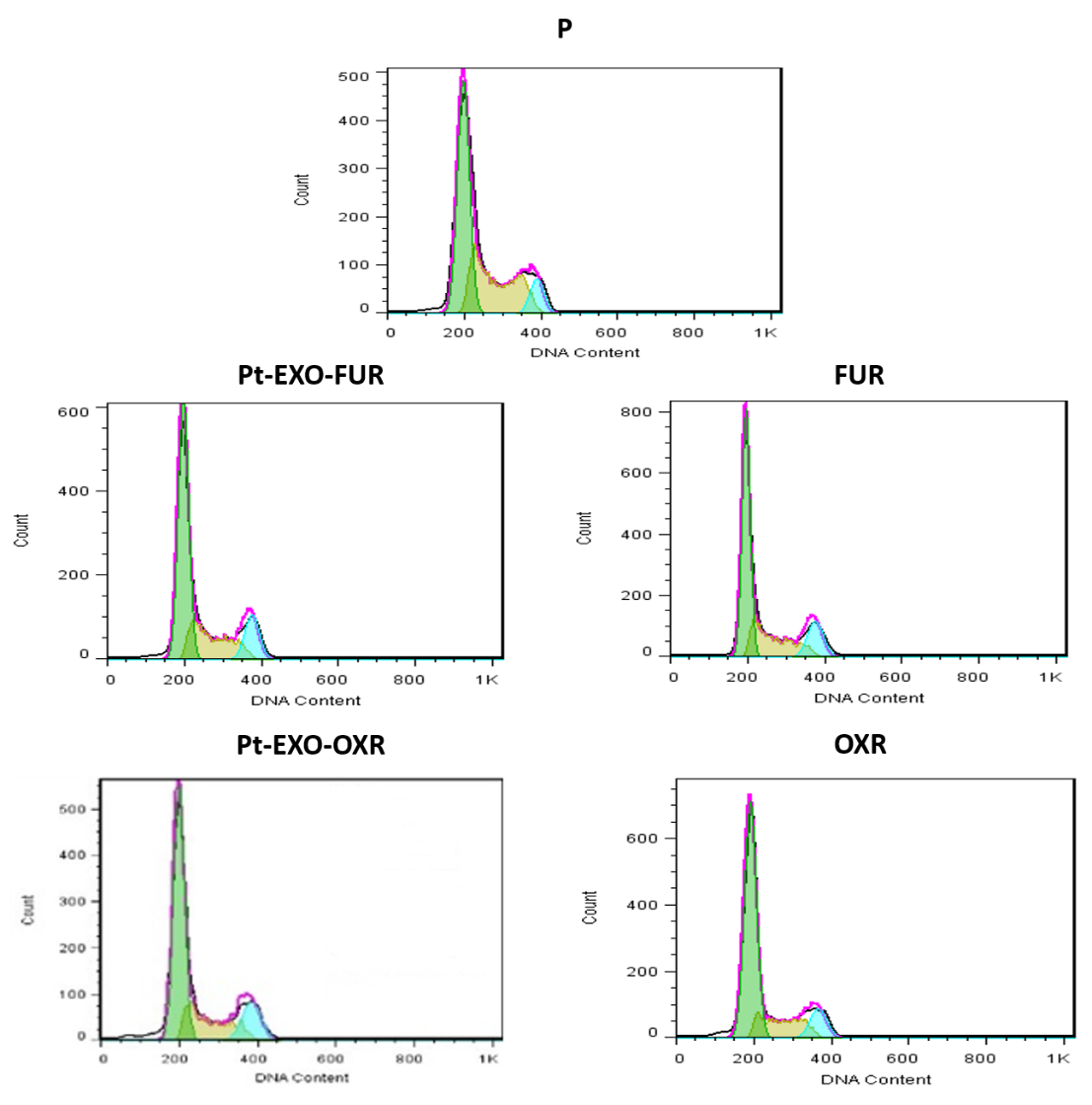


**Fig. S2** The cell cycle was analyzed by flow cytometry in 5-FU and oxaliplatin resistance. The graphs represent the cell cycle in Parental cells (P), 5-FU resistant cells (FUR), oxaliplatin resistant cells (OXR), and parental cells treated with exosomes derived FUR (Pt-EXO-FUR) and OXR (Pt-EXO-OXR) cells after 72 h.


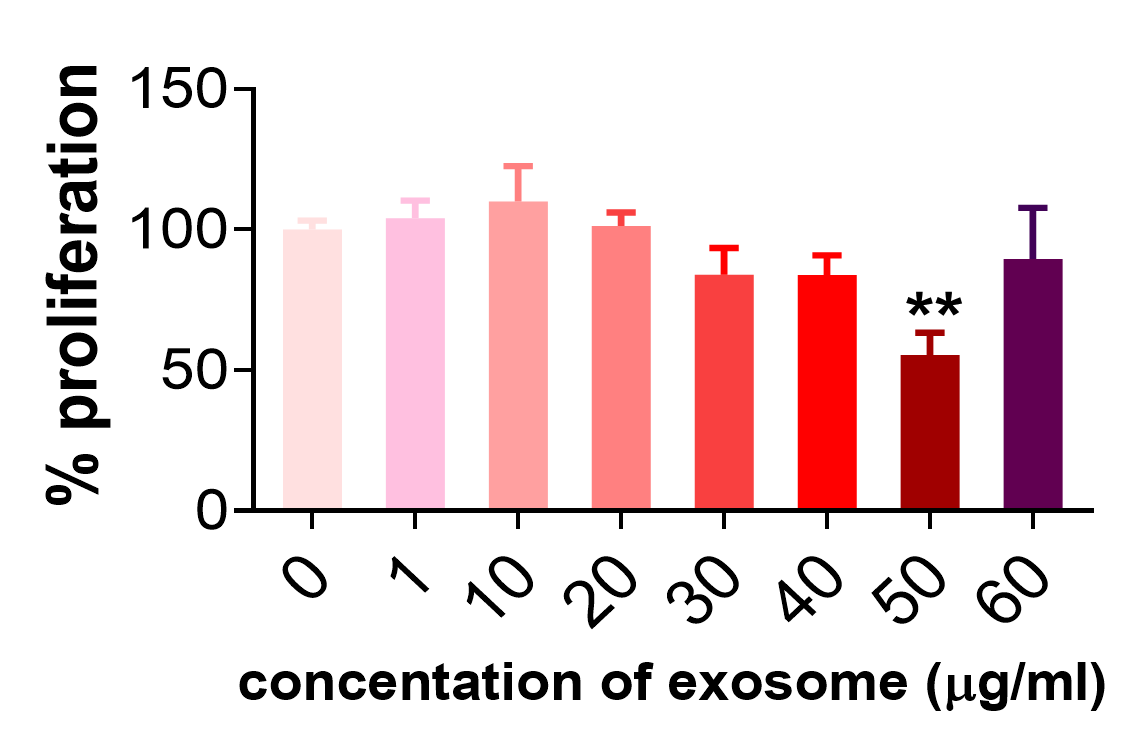


**Fig. S3** Effects of parental cell exosomes on proliferation of parental cells. Parental cells were treated autologously with different concentrations of exosomes secreted from parental cells (EXO-P) and after 72 h, cell viability was assessed by MTT assay.


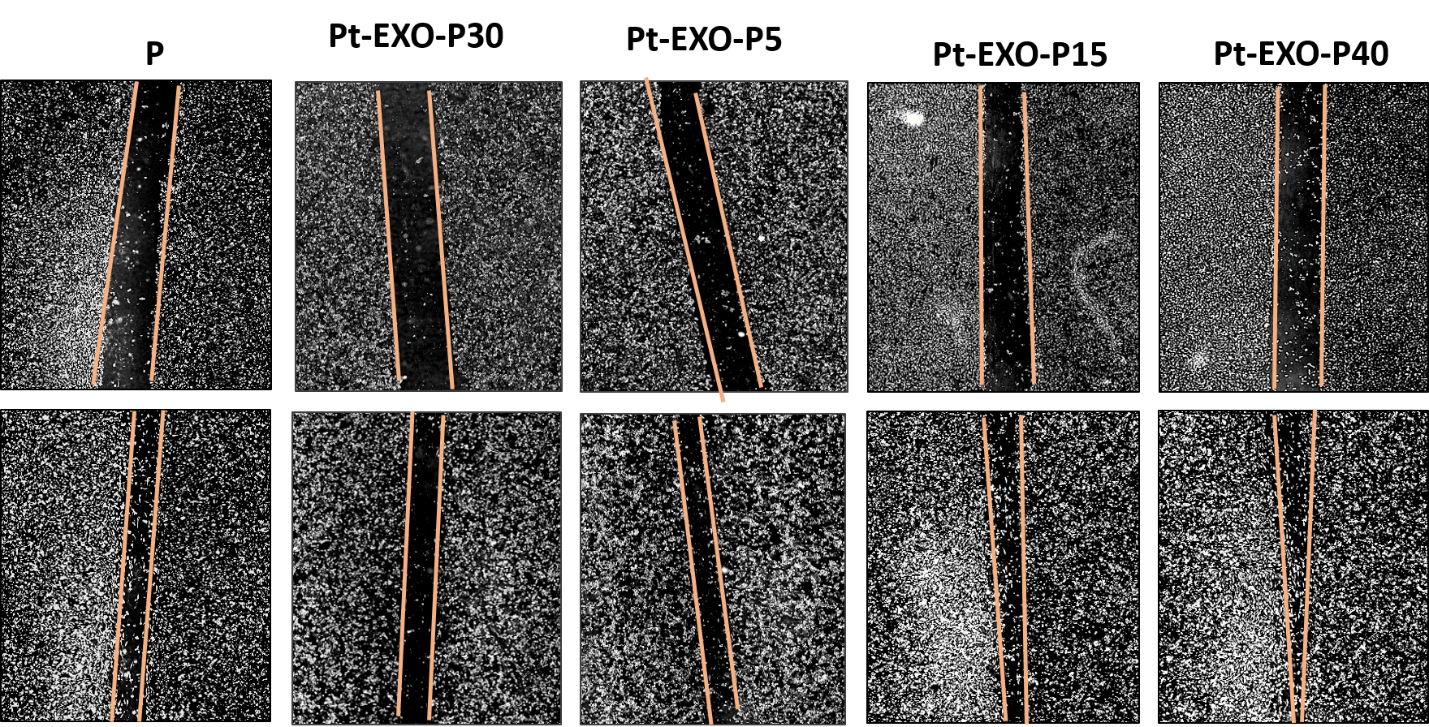


**Fig. S4** The impact of oxaliplatin and 5-FU treatment on parental cell migration via exosomes. The images show the effects of exosomes from 5 or 30 μM oxaliplatin-treated parental cells (EXO-P5 or EXO-P15) or 15 or 40 μM 5-FU-treated parental cells on migration of parental cells.
